# Supplementary material for: Efficient Global MOT Under Minimum-Cost Circulation Framework
Source: IEEE Trans Pattern Anal Mach Intell. Author manuscript; Available in PMC 2022 Apr 1. (PMC8966209; doi:10.1109/TPAMI.2020.3026257)
Supplement: supp1-3026257 [file NIHMS1786283-supplement-supp1-3026257.pdf]

Efficient Global Multi-object Tracking Under Minimum-cost Circulation Framework
# - Appendices -

Congchao Wang, Yizhi Wang, Guoqiang Yu

The Bradley Department of Electrical and Computer Engineering, Virginia Tech

1 Details about experiments

1.1 Implementation details

SSP and dSSP are both implemented in C++. The key step for these two methods is the implementation of the Dijkstra's algorithm for the shortest path searching. We use the data structure of self-balanced binary search tree to implement the Dijkstra's algorithm which uses $O(1)$ time for popping the top element and $O(\log(n))$ for pushing a new element.

All comparisons were conducted on Ubuntu 16.04 LTS with the code compiled by g++ v5.4.0. The CPU is a 2.40GHz Xeon(R) CPU E5-2630, but only a single core is used. The RAM size is 128GB and the memory speed is 2133MHz.

1.2 Specs of the videos and graphs

The details of the datasets we used in the experiments can be found in Table 1, 2, and 3. For each dataset we show in the table the number of frames and the number of detections in each video. The out-coming graph sizes with respect to different affinity models are also listed. Notice that the arc numbers can be different with different affinity models. The numbers listed

Table 1: Details of KITTI-Car dataset

Datasets	KITTI(DPM)				KITTI(reglets)			
	seq00	seq10	seq11	seq14	seq00	seq10	seq11	seq14
#frames	465	1176	774	850	465	1176	774	850
#detections	51100	181132	104748	96974	19885	22189	24524	35198
(a) graph design from [1]								
#vertices	102201	362265	209497	193949	39771	44379	49049	70397
#arcs	171135	608881	349869	325256	71730	78273	86864	123008
(b) graph design from [2]								
#vertices	102201	362265	209497	193949	39771	44379	49049	70397
#arcs	173242	609576	352140	328352	71977	78524	87048	122875
(c) graph design from [3]								
#vertices	102201	362265	209497	193949	39771	44379	49049	70397
#arcs	172317	607035	350276	326635	71592	78477	86830	122763

Table 2: Details of CVPR19 and EHTZ datasets

Datasets	CVPR19				ETHZ	
	seq04	seq06	seq07	seq08	seq03	seq04
#frames	2080	1008	585	806	1000	936
#detections	208000	70189	20220	43444	101180	94054
(a) graph design from [1]						
#vertices	416001	140379	40441	86889	202361	188109
#arcs	9522469	3343504	759961	1866996	358546	365461
(b) graph design from [2]						
#vertices	416001	140379	40441	86889	202361	188109
#arcs	9712807	3703396	1001361	2076685	411170	368080

Table 3: Details of PTC and Embryo datasets

	#frames	#detections	#vertices	#arcs
PTC-High	101	77352	154705	462213
PTC-Mid	101	39215	78431	234438
PTC-Low	101	7438	14877	44448
Embryo	531	6750628	13501257	60378108

in the tables correspond to the graphs used in the minimum-cost circulation-based framework. For any graph used in the minimum-cost flow-based framework, the number of vertex should be subtracted by one compared with its corresponding graph used in the minimum-cost circulation-

based framework (for more details, please see section 4 of the main paper).

References and Notes

[1] H. Pirsiavash, D. Ramanan, and C. C. Fowlkes, "Globally-optimal greedy algorithms for tracking a variable number of objects," in *CVPR 2011*. IEEE, 2011, pp. 1201–1208.

[2] P. Lenz, A. Geiger, and R. Urtasun, "Followme: Efficient online min-cost flow tracking with bounded memory and computation," in *Proceedings of the IEEE International Conference on Computer Vision*, 2015, pp. 4364–4372.

[3] S. Sharma, J. A. Ansari, J. K. Murthy, and K. M. Krishna, "Beyond pixels: Leveraging geometry and shape cues for online multi-object tracking," in *2018 IEEE International Conference on Robotics and Automation (ICRA)*. IEEE, 2018, pp. 3508–3515.
